# Meta-analysis of broad-spectrum antibiotic therapy in patients with active inflammatory bowel disease

**DOI:** 10.3892/etm.2012.718

**Published:** 2012-09-20

**Authors:** SHENG-LAN WANG, ZHI-RONG WANG, CHANG-QING YANG

**Affiliations:** Division of Gastroenterology and Digestive Diseases Institute, Tongji Hospital of Tongji University School of Medicine, Shanghai 200065, P.R. China

**Keywords:** antibiotic therapy, Crohn’s disease, ulcerative colitis

## Abstract

Patients with Crohn’s disease (CD) or ulcerative colitis (UC) undergo various therapies, including antibiotic therapy. This meta-analysis of controlled clinical trials was conducted to evaluate whether the use of antibacterial therapy improves the clinical symptoms of inflammatory bowel disease (IBD). The Medline and Scopus databases were searched and a systematic review was performed. Randomized, controlled trials in which antibiotic therapy was compared with placebo were investigated. A total of 10 randomized, placebo-controlled clinical trials for CD were included in the meta-analysis. The pooling of the data from these trials yielded an odds ratio (OR) of 1.35 [95% confidence interval (CI), 1.16–1.58] for antibiotic therapy compared with placebo in patients with CD. Furthermore, nine randomized placebo-controlled clinical trials for UC matched our criteria and were included in the analysis. The pooling of the data from these trials yielded an OR of 2.17 (95% CI, 1.54–3.05) in favor of antibiotic therapy. These results suggest that antibiotics improve clinical outcomes in patients with IBD.

## Introduction

Crohn’s disease (CD) and ulcerative colitis (UC) are covered by the classification of inflammatory bowel disease (IBD). IBD is an idiopathic disease resulting in intestinal mucosal inflammation and loss of the barrier function. The exact mechanisms of the induction and development of IBD are not yet fully understood. Multiple factors may contribute to this condition. An immunological disorder coupled with intolerance to intestinal flora appear to be the most significant. In certain cases, they are associated with gene mutations induced by environmental factors or pathogenesis. The terminal ileum and colon have a high bacterial content, which in IBD stimulates inflammation in the intestinal lumen (1). A study of IBD patients revealed that the loss of immune tolerance to symbiotic bacteria involved increased T-cell and humoral immune responses (2). The chronic idiopathic IBDs, CD and UC, appear to involve an over-active immune response to bacterial antigens in genetically susceptible individuals (3). The therapy of IBDs typically comprises immunosuppressive drugs, but clinicians tend also to use antibiotics as certain symptoms, including fever, purulent stools, abscesses and other signs of infection, have been considered to be caused by bacteria (1).

Although scientists have debated whether bacteria are the primary cause of CD, they cause only a superimposed bacterial infection of the lesions while the disease is caused by an overaggressive immune response to the bacteria. Several studies have examined the effects of anti-tuberculosis treatment on IBD, but these trials have generated conflicting results. Animal data support the anti-inflammatory effects of a small number of antibiotics, while in studies of CD, anti-inflammatory effects have been shown in patients using metronidazole and ciprofloxacin (4). However, there is no definite proof of systemic antibiotics as the main treatment due to lack of valid data on CD.

The objective of this meta-analysis was to perform a systematic review and meta-analysis of randomized, placebo-controlled clinical trials to assess the effectiveness of antibiotic therapy in patients with CD.

## Materials and methods

### Literature search

Only randomized, controlled trials which compared antibiotic therapy to placebo in patients with CD or UC were included. We searched the Medline and Scopus databases from 1970 to 2010 using keywords that denote IBDs, antibacterial and antimycobacterial drugs, antibacterial activity, metronidazole and ciprofloxacin. The language used was English.

### Data abstraction

The standardized data abstraction form and main outcome data, including treatment programs, sample size and results, were selected by two independent observers.

Ethambutol, isoniazid and rifamycins (including rifampicin and rifabutin) were considered to be classic drugs against infection with *Mycobacterium tuberculosis*, and trials of antituberculosis drugs were included in the meta-analysis. Similarly, nitroimidazoles (metronidazole), macrolides (clarithromycin) and riminophenazines (clofazimine), were also analyzed together (Tables I and II).

### Statistical analysis

Homogeneity was assessed using a χ^2^ test of homogeneity and the graphics were displayed simultaneously. A pooled estimate of the odds ratio (OR) was calculated and applied to a Mantel-Haenszel method test. Study results are presented as ORs with 95% confidence intervals (CIs). For studies with continuous outcome measures, results were converted to ORs. The log OR corresponds to a constant multiplied by the standardized difference between means.

## Results

**Figure 1:**
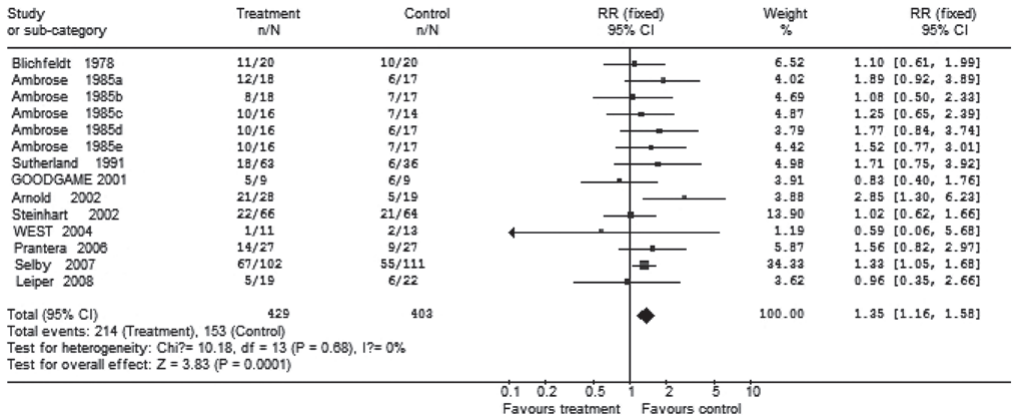
Analysis of trials based on the use of antibiotic therapy with or without a tapering course of corticosteroids in Crohn’s disease. RR, risk ratio; CI, confidence interval.

**Figure 2:**
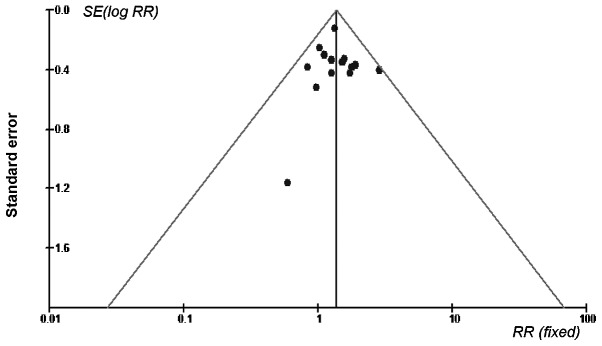
Funnel plot of indicators of bias for the outcome of clinical improvement in studies of antibiotic therapy for Crohn’s disease. RR, risk ratio.

All included studies had a double-blind design. The trials involved a total of 832 patients with CD who were randomized to receive broad-spectrum antibiotic therapy; 429 patients were treated with antimicrobials while 403 patients received placebo. In the antibiotic group, 39 (9.1%) received ciprofloxacin, 135 (31%) patients received metronidazole alone, 32 (7.4%) received cotrimoxazole alone, 19 (4.4%) patients received clarithromycin alone and 66 (15.3%) patients received metronidazole plus ciprofloxacin. In these trials, some patients received concomitant therapy and some did not. Clinical improvement occurred in 56.1% (214/429) of patients in the antibiotic group and 37.9% (153/403) of patients in the placebo group. The summary OR for clinical improvement with any antibiotic therapy in the trial was 1.35 (95% CI, 1.16–1.58), The Breslow-Day test for heterogeneity indicated that the studies were not significantly heterogeneous (Figs. 1 and 2).

**Figure 3:**
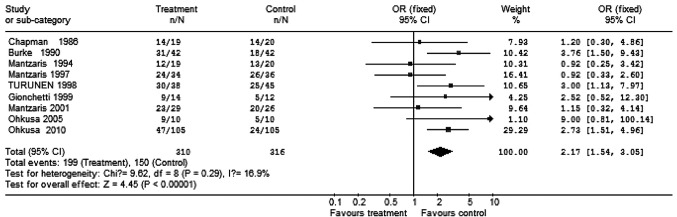
Individual and pooled odds ratios (ORs) for clinical remission in studies that considered antibiotic therapy in ulcerative colitis. CI, confidence interval.

**Figure 4:**
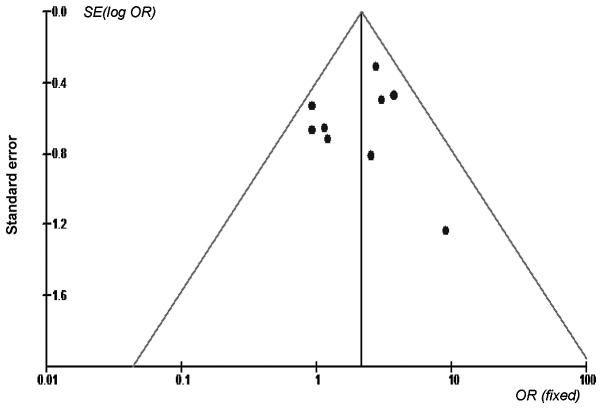
Funnel plot of indicators of bias for the outcome of clinical improvement in studies of antibiotic therapy for ulcerative colitis. OR, odds ratio; CI, confidence interval.

A total of 626 patients taking part in trials for UC were randomized to receive antibiotics; 310 patients were treated with antibiotics and 316 patients received placebo. In those who received antibiotics, 101 (32.6%) received ciprofloxacin, 115 (37.1%) received amoxicillin plus tetracycline plus metronidazole, 42 (13.5%) received tobramycin alone and 19 (6.1%) received metronidazole alone. Remission was induced in 64.2% of the patients treated with antibiotics, compared with 47.5% of the placebo group. The pooling of these trials yielded an OR of 2.17 (95% CI, 1.54–3.05) in favor of antibiotic therapy. The Breslow-day test for heterogeneity revealed that the studies were statistically homogeneous and may be combined (Figs. 3 and 4).

## Discussion

### Crohn’s disease

The majority of this work has been conducted using metronidazole, ciprofloxacin or clarithromycin alone or in combination. The two antibiotics most commonly used in the treatment of CD are metronidazole and ciprofloxacin. These antibiotics are active against two of the bacterial species suspected to be involved in the pathogenesis and, at least, relieve the symptoms of CD. Their use is widely accepted for the treatment of perianal fistula and colitis, although there is incontrovertible evidence supporting their use for these conditions.

The few randomized controlled trials to study metronidazole and/or ciprofloxacin have mostly presented negative results (5), although the treatment has been reported to be more effective in those patients whose disease involved the colon (6,7).

One study made use of rifaximin, a non-absorbed rifamycin drug with a broad spectrum of activity against Gram-positive, Gram-negative and even colonic anaerobic bacteria. Owing to the fact that it is not absorbed, systemic adverse effects are rare. In a double-blind, placebo-controlled trial reported by Prantera *et al* (8), 83 CD patients were divided into 3 groups: group A received rifaximin, 800 mg once a day; group B received rifaximin, 400 mg twice daily; and group C received placebo. Remission rates were significantly higher (52%) in group B than in group A (32%) and in the placebo group (33%) (8). Thus, the antibiotics with cell activity, including the macrolide antibiotics azithromycin and clarithromycin, may provide a more effective chemotherapy. Based on the role of intestinal microflora in the pathogenesis of CD, the use of antibiotics or combined therapy, appears to be a rational strategy. In a previously published study, ciprofloxacin was shown to be effective when added to the existing medium-active treatment in therapy-resistant CD patients (9). Ciprofloxacin, a quinolone drug, is clinically effective in preventing the growth of intestinal bacteria and appears to have immunomodulatory properties. Accumulating evidence supports its role as the primary treatment for CD. For example, in a prospective study by Colombel *et al* (16), ciprofloxacin (500 mg twice daily) was as effective as mesalazine (4 g/day) and induced remission in patients with mild to moderate CD.

Two important observations have strengthened the bacterial hypothesis for CD. Firstly, genetic studies have identified mutations in the NOD2 gene and the IRGM and ATG16L1 autophagy genes (17). These mutations mean that cells do not contain intracellular bacterial replication, and may have defects in their ability to eliminate bacteria (18). Secondly, an increased number of mucosa-associated *E. coli* have been identified in patients with IBD (19).

In addition to additional data being required to confirm the effectiveness of antibiotics in the treatment of CD, there is the emergence of resistant bacterial strains and the possible infection by *Clostridium difficile* to be considered. A study has reported the previous use of antibiotics by the majority of a group of IBD patients with *Clostridium difficile* infection (20). In an uncontrolled trial, antibiotic therapy resulted in clinical remission in 49 cases (68%) (21). In the same study, 55 patients (76%) showed a clinical response, which was higher in those patients who were also taking steroids (26/29) than those individuals who were not (29/43).

A large amount of data support the use of antibiotics in patients with CD and with diverticulitis. Antibiotics may act via different pathways in patients with CD. They may inhibit the bacteria linked to the pathological processes of the disease or simply lower the luminal bacterial overgrowth. The suppression of intestinal flora may reduce the strength of certain symptoms, including pain and diarrhea.

In our analysis, CD patients undergoing antibacterial therapy are 1.35-fold more likely to experience clinical remission than patients who are not undergoing antibacterial therapy. Homogeneity among the trials was established based on a graphic display and statistical tests. In addition, the meta-analysis of short-term antimicrobial use also revealed that treatment with antibiotics was effective in the induction of clinical remission. These results were clinically and statistically significant.

Whether antibiotics are of actual benefit remains to be determined since only a few controlled trials have been completed. Many used a small number of volunteers or used subgroups which were not always clearly defined. Antibiotic use has predominantly involved metronidazole or ciprofloxacin, and many patients cannot tolerate these drugs for a long duration. Therefore, these antibiotics are used in the treatment of ileal, ileocolonic and colonic CD (22).

### Ulcerative colitis

Several different antibiotics, alone and in combination, have been evaluated in the primary and adjuvant treatment of active UC. The routine use of antibiotics is not recommended in mild or moderate UC. In a prospective, double-blind, randomized controlled trial reported by Gilat *et al* (23), oral metronidazole and sulfasalazine were used for the outpatient management of mild to moderate UC. This revealed that metronidazole was significantly less effective than sulfasalazine by endoscopic and clinical criteria.

In a randomized controlled trial reported by Chapman *et al* (24), 39 patients with severe UC were treated with either intravenous metronidazole (500 mg every 8 h) or placebo as an adjunct to intravenous steroids for 5 days. No significant difference was observed in clinical improvement between the two groups. In two randomized controlled trials reported by Mantzaris *et al* (25,26), patients with mild to severe acute UC received either oral or intravenous ciprofloxacin for 2 weeks as an adjunct to corticoid therapy. No significant difference in clinical improvement was observed, with 71% (24/34) and 79% (23/29) of the patients in the ciprofloxacin-treated group and 72% (26/36) and 77% (20/26) of the patients in the placebo-treated group achieving remission. The authors concluded that a short course of oral ciprofloxacin treatment did not appear to increase the proportion of patients with active UC going into remission. In another study, the combination of intravenous metronidazole and tobramycin as an adjunct to corticosteroids was not found to be effective in causing clinical improvement compared with placebo after 10 days of therapy in the treatment of severe UC (27).

However, antibiotics may have a certain benefit as adjuncts to standard anti-inflammatory treatment. Burke *et al* (28) reported a study in which 84 cases of acute UC were randomized to receive oral tobramycin or placebo for 7 days as an adjunct to steroidal therapy. When evaluated 18–21 days after the end of treatment, 31/42 patients (74%) in the tobramycin group and 18/42 patients (43%) in the placebo group had achieved clinical remission (p=0.008).

Despite considerable evidence for the involvement of bacteria in UC, broad-spectrum antibiotics are usually not used for the treatment of this disease; severe cases are the exception. Only a few antibiotics have been used in the treatment of UC, whether individually or in combination. One trial has shown that rifaxamin is beneficial in the treatment of UC (29). Turunen *et al* (30) performed a randomized, controlled trial which suggested that ciprofloxacin is beneficial as an adjunctive treatment for active UC during the first 6-month period of administration. At 6 months, the treatment-failure rate in the ciprofloxacin-treated group was 21% (8/38 patients), which was significantly lower than the placebo group rate of 44% (20/45 patients). The authors concluded that the use of ciprofloxacin therapy for 6 months in UC improved the results of conventional treatment with mesalazine and prednisone.

In our study, UC patients undergoing antibacterial therapy have been determined to be 2.17-fold more likely to experience clinical remission than patients receiving no antibacterial therapy. Homogeneity among the trials was established based on a graphic display (Fig. 2) and statistical tests. In addition, meta-analysis of short-term antibacterial trials revealed that 5–180 days of antibiotic therapy is effective for clinical remission. These results were clinically and statistical significant.

However, a lack of well-designed, placebo-controlled trials has made the efficacy of antibiotics as the primary treatment for IBD questionable. Poor study design, high drop-out rates and insufficient numbers of subjects in the current studies have led to negative or equivocal results causing further controversy.

To date, these studies have provided sufficient evidence that antibiotics have been useful in the treatment of this disease. The use of antibiotics in patients with IBD, however, is controversial. The treatment of experimental IBD with antibiotics may have contradictory results, since it remains unclear which bacteria cause the disease. Moreover, many organisms which grow in the gut mucosa, where they are more resistant to standard antibiotic treatment, may not be able to reach the mucosal surface. Susceptibility testing of organisms has not been carried out in a systematic manner, and antibiotic treatment is not aimed at specific bacteria. In fact, other clinical infections may be present. It would be a difficult and time-consuming task to complete in clinical practice if multiple varieties of bacteria were involved. This is due to the requirement for the mucosal microbial flora of each patient to be characterized and a drug sensitivity test on each patient to be carried out.

Therefore, to determine the effect of antibiotics in the treatment of IBD, larger randomized clinical trials of antibiotics should be carried out, either in solo or combined with other antibiotics or therapies.

## Figures and Tables

**Table I:** Summary of randomized, controlled trials included in the meta-analysis of Crohn’s disease.

Author (ref)	Mean age	Gender (M/F)	Regimens	Concomitant therapy	Duration	Clinical improvement	
						Antibiotics	Placebo
Arnold *et al*, 2002 (9)	45.2	28/19	Cipro 500 mg b.i.d.	Prednisone	4 weeks	21/28	5/19
Prantera *et al*, 2006 (8)	38±12	16/11	Rifaximin 800 mg b.i.d.	Aminosalicylate	12 weeks	14/27	9/27
West *et al*, 2004 (10)	34	12/12	Cipro 500 mg b.i.d.	Infliximab	6 weeks	1/11	2/13
Steinhart *et al*, 2002 (7)	32	57/77	Cipro 500 mg b.i.d. + metro 500 mg b.i.d.	Budesonide	8 weeks	22/66	21/64
Leiper *et al*, 2008 (11)	34	17/24	Clari 1 g/day		12 weeks	5/19	6/22
Goodgame *et al*, 2001 (12)	39.4±9.2	18/13	Clari 500 mg b.i.d. + ethambutol 15 mg/kg		12 weeks	5/9	6/9
Blichfeldt *et al*, 1978 (13)	27.5	8/12	Metro 250 mg q.i.d.	Prednisone	8 weeks	11/20	10/20
Ambrose *et al*, 1985 (14)	36.5	13/22	Metro 400 mg b.i.d.		2 weeks	12/18	6/17
					4 weeks	8/18	7/17
					6 weeks	10/16	7/14
Ambrose *et al*, 1985 (14)	37.0	12/21	Sulfa 960 mg b.i.d.		2 weeks	10/16	6/17
					4 weeks	10/16	7/17
Sutherland *et al*, 1991 (6)	NA	NA	Metro 10–20 mg/kg/day		16 weeks	18/63	6/36
Selby *et al*, 2007 (5)	36.5±11.3	101/112	Clari 750 mg/day + rifampicin 450 mg/day + clofa 50 mg/day	Prednisone	16 weeks	67/102	55/111

Cipro, ciprofloxacin; metro, metronidazole; clari, clarithromycin; sulfa, sulfamethoxazole; clofa, clofazimine; NA, not available.

**Table II:** Characteristics of studies included in the meta-analysis of ulcerative colitis.

Author (ref)	Mean age	Gender (M/F)	Regimens	Concomitant therapy	Duration	Clinical improvement	
						Antibiotics	Placebo
Burke *et al*, 1990 (28)	43.5	28/19	Tobra 120 mg t.i.d.	Corticosteroids	7 days	31/42	18/42
Ohkusa *et al*, 2005 (31)	39.5	12/8	Amoxi 500 mg t.i.d. + Tetra 500 mg t.i.d. + metro 250 mg t.i.d.	Aminosalicylate + corticosteroids	14 days	9/10	5/10
Mantzaris *et al*, 2001 (26)	41.5	26/29	Cipro 400 mg b.i.d.		10 days	23/29	20/26
Mantzaris *et al*, 1997 (25)	41.5	33/37	Rifaximin 400 mg b.i.d.	Corticosteroids	14 days	24/34	26/36
Gionchetti *et al*, 1999 (29)			Cipro 500–750 mg b.i.d.		10 days	9/14	5/12
Turunen *et al*, 1998 (30)	34.2	58/25	Cipro 500–750 mg b.i.d.	Corticosteroids Aminosalicylate	180 days	30/38	25/45
Ohkusa *et al*, 2010 (32)	NA	NA	Amoxi 500 mg t.i.d. + tetra 500 mg t.i.d. + metro 250 mg t.i.d.		90 days	47/105	24/105
Chapman *et al*, 1986 (24)	46.0	19/20	Metro 500 mg t.i.d. i.v.	Prednisone	5 days	14/19	14/20
Mantzaris *et al*, 1994 (27)	NA	NA	Metro 0.5 g t.i.d. i.v. + tobra 4 mg/kg t.i.d.	Hydrocortisone	10 days	12/19	13/20

Tobra, tobramycin; amoxi, amoxicillin; tetra, tetracycline; metro, metronidazole; cipro, ciprofloxacin; NA, not available.
